# Nutrition Policy and Programs in Educational Settings: Equitable Approaches to Food Security

**DOI:** 10.3390/nu18050773

**Published:** 2026-02-27

**Authors:** Sarah Moreland-Russell, Dan Ferris

**Affiliations:** 1 School of Public Health, Washington University in St. Louis, St. Louis, MO 63130, USA; 2 Brown School, Washington University in St. Louis, St. Louis, MO 63130, USA; dan.ferris@wustl.edu

## 1. Introduction

Food insecurity is a persistent determinant of poor health and unequal educational outcomes, particularly among children and young people experiencing racial and economic inequities. Research has demonstrated the negative effects of food insecurity on health and wellbeing [1,2], development [3], and academic performance [4,5,6,7]. Educational settings including childcare, primary and secondary schools, and universities, are uniquely positioned to reduce nutrition-related disparities through policies and programs that increase access to healthy foods and shape lifelong dietary behaviors. Educational settings matter both because of the evidence that hunger impedes learning and, from an intervention and implementation viewpoint, because of their immense reach and role not only for individual students but as anchors of larger communities.

In the U.S., the National School Lunch and School Breakfast Programs serve over 30 million students lunch and 15 million students breakfast in more than 90,000 schools and institutions [8]. Leveraging educational settings to support child and family food security is not unique to the U.S., exemplified by the variety of models and approaches around the world [9]. Globally, the estimated number of children who participate in school meal programs (466 million) reflects an increase of 20% between 2020 and 2024 [10,11,12,13].

This Special Issue, entitled “Nutrition Policy and Programs in Educational Settings: Equitable Approaches to Food Security”, was developed to advance evidence on the design, testing, evaluation, implementation, and dissemination of interventions and policies that explicitly prioritize nutritional equity in educational contexts. The seven papers published in this Special Issue span multiple global geographies and methodological approaches, offering complementary perspectives on (1) how school food policy is governed and implemented; (2) how different actors shape the feasibility and acceptability of nutrition standards (e.g., interest groups, distributors, parents, and school food administrators); and (3) how research can more effectively measure equitable policy implementation.

## 2. Overview of the Published Articles

This Special Issue presents scholarship that reflects the breadth of educational nutrition policy, from national rights-based feeding programs and global evidence mapping to local implementation supports and debates over meal standards.

*Program evaluation and outcomes in practice (Australia).* Smith et al. examine whether the 2022–2023 Tasmanian School Lunch Project, which provided free nutritious cooked lunches in schools serving high-disadvantage communities, was associated with improved attendance. Although school staff perceived multiple benefits, the study did not find an association between the program and grade-level attendance when assessed using administrative data and comparison schools—reinforcing the importance of selecting outcomes that reflect intervention pathways and adopting rigorous evaluation designs for complex real-world programs [14].

*Governance, rights-based policy, and social control in school feeding systems (Brazil).* Hartmann et al. provide a review and content analysis of Brazilian social documents and legislation related to the National School Feeding Program. The authors situate school feeding within Brazil’s Food and Nutrition Security policies and the human right to adequate food, underscoring that democratic accountability mechanisms, particularly social control structures, are essential for ensuring compliance and corrective action to maintain program objectives [15].

*Global research trends and future directions for free school lunch policy (Indonesia-focused).* Abadi et al. combine bibliometric analysis with critical review to map international research and identify emerging priorities for free school lunch programs. The authors report increasing attention to sustainability, food waste management, and nutrition education, while highlighting continuing disparities in access and implementation. They propose a future-oriented framework emphasizing stakeholder collaboration, culturally adaptive design, and technology-enabled innovations to strengthen nutrition policy and broader development goals [16].

*Food insecurity, development, and the role of childcare settings (United States).* Casey and Winsler synthesize evidence on food insecurity and child development and argue for strengthening the role of childcare as a critical nutrition and equity intervention setting. By extending attention to early childhood systems, this contribution underscores the importance of intervening across the educational lifespan, particularly during sensitive developmental periods where nutrition-related risk can compound disparities in health and learning [17].

*Urban–rural inequities in wellness policy environments (United States).* Iyer et al. investigate differences between urban and rural school districts’ wellness policies and implementation infrastructure. Findings indicate that rural districts had fewer wellness policy goals and lower odds of implementation supports such as wellness plans and school health advisory councils, highlighting the need for tailored technical assistance and resourcing approaches that prevent geography-based inequities in policy uptake and sustainability [18].

*Equity-centered measurement development to strengthen implementation science (United States).* McLoughlin et al. address a critical gap in school health policy research: the limited availability of measures that capture equitable implementation processes and outcomes. Their two-year measurement development study identifies key equity-focused constructs and advances survey instruments informed by expert consultation and cognitive testing, supporting stronger assessment of “how” and “for whom” school nutrition policies achieve intended effects [19].

*Stakeholder perspectives and policy flexibility in school meal standards (United States).* Moreland-Russell et al. analyze public comments submitted in response to proposed USDA flexibilities affecting milk, whole grains, and sodium requirements. Their longitudinal qualitative content analysis illustrates how stakeholder groups vary in their support for policy change and emphasizes that shifts in nutrition standards are shaped not only by evidence, but also by feasibility constraints, values, and political dynamics [20].

## 3. Future Research

Taken together, the papers in this Special Issue underscore that across diverse geographies, achieving nutritional equity in educational settings requires action across governance, implementation capacity, evaluation, and measurement. Several directions emerge for future work.

First, equity must be operationalized in implementation, not only articulated in policy goals. Evidence-based and equity-centered approaches to implementing nutrition standards should be reinforced by financing, monitoring, and accountability systems that prevent uneven benefits across population groups and settings. Second, evaluation frameworks should incorporate outcomes aligned with plausible pathways of effect (e.g., diet quality, stigma reduction, classroom readiness) alongside education outcomes such as attendance. Third, equity-centered measurement development remains essential for identifying why policies succeed in some contexts and stall in others, enabling scalable and sustainable strategies for program improvement.

Finally, cross-cutting engagement and feasibility work is needed to ensure that nutrition standards remain both evidence-based and operationally achievable in diverse school environments. Addressing these priorities will strengthen the translation of research into policy and practice, supporting school and childcare nutrition systems that reduce disparities and improve child and youth well-being.
